# Immune Responses Induced by Recombinant *Bacillus subtilis* Expressing the PEDV Spike Protein Targeted at Microfold Cells

**DOI:** 10.3390/vetsci9050211

**Published:** 2022-04-25

**Authors:** Jian Lin, Chunxiao Mou, Shuai Zhang, Liqi Zhu, Yuchen Li, Qian Yang

**Affiliations:** College of Veterinary Medicine, Nanjing Agricultural University, Weigang 1, Nanjing 210095, China; linjian@njau.edu.cn (J.L.); 006810@yzu.edu.cn (C.M.); 2017207006@njau.edu.cn (S.Z.); zhuliqi@yzu.edu.cn (L.Z.); yuchengli@njau.edu.cn (Y.L.)

**Keywords:** *Bacillus subtilis*, M cells, PEDV S protein, oral immunisation

## Abstract

*Bacillus subtilis* (*B. subtilis*), a probiotic bacterium and feeding additive, is widely used for heterologous antigen expression and protective immunisation. Porcine epidemic diarrhoea virus (PEDV) invades swine via mucosal tissue. To enhance the mucosal immune response to PEDV, we modified *B. subtilis* to express a PEDV antigen and used it as a mucosal vaccine delivery system. Initially, we constructed a recombinant *B. subtilis* strain (*B.s-RCL*) that expressed the PEDV spike protein and L-Lectin-β-GF, with the goal of inducing mucosal secretory immunoglobulin A (sIgA) and anti-PEDV serum immunoglobulin G (IgG) production, as well as to increase the number of microfold cells (M cells). Following the oral administration of *B.s-RCL* to mice, the small intestinal PEDV-specific sIgA expression levels significantly increased, as well as the increased number of *B.s-RCL* adhered to M cells. Moreover, we found that mice administered *B.s-RCL* exhibited markedly higher percentages of CD4^+^ and CD8^+^ T cells in the mesenteric lymph nodes and spleen compared to the control mice. Furthermore, we found that intestinal mucosa sIgA and serum anti-PEDV IgG levels were higher in mice orally immunised with *B.s-RCL*, suggesting that the mice could be more resistant to PEDV. In this study, we developed a novel oral vaccine to prevent porcine diarrhoea epidemics.

## 1. Introduction

*Bacillus subtilis* (*B. subtilis*) is a non-pathogenic Gram-positive bacterium that is widely used as a probiotic. *B. subtilis* is used as a feed additive to improve the intestinal microbiome [1] and as a mucosal adjuvant to improve mucosal immunity [2,3,4]. Furthermore, *B. subtilis* has a stable heterologous protein expression system and is commonly used as a vehicle for antigen expression [5,6]. In our previous study, we developed a recombinant *B. subtilis* strain that could induce an immune response to the transmissible gastroenteritis virus via dendritic cell uptake [7]. However, due to the limited number of dendritic cells in the intestinal mucosa, *B. subtilis* exhibited poor adhesion to intestinal epithelial cells [8]. This limited the uptake of *B. subtilis* by intestinal dendritic cells, leading to poor activation of the mucosal immune response. Therefore, a novel antigen delivery vector that can trigger an immune response against mucosal pathogens is needed [9].

Normally in gastrointestinal mucosa, the immunologically active sites are located in Peyer’s patches [10]. Besides dendritic cells, microfold cells (M cells) are another important intestine cell located on the surfaces of Peyer’s patches, where they are involved in antigen uptake, transport and processing [9]. M cells transfer antigens to various immune cells, such as dendritic cells, T cells, B cells and macrophages, thus inducing an immune response [11,12]. In a previous study, recombinant yeast expressing M cells effectively invaded the mucosa [9]. The adhesion protein L-lectin was first discovered in leguminous seeds, where it binds to extracellular sugar chains [13]. Various types of L-lectin proteins can recognise different carbohydrates, despite having similar folding structures [14]. The surface protein L-Lectin-β-GF mediates the adhesion of *Staphylococcus aureus* to M cells [15]. β-GF binds to secretory immunoglobulin A (sIgA), which is then taken up by M cells in the intestinal mucosa [16]. Therefore, we hypothesised that the M cell-targeting L-Lectin-β-GF protein may improve the adherence of *B. subtilis* to M cells.

Porcine epidemic diarrhoea virus (PEDV) causes severe enteric disease in pigs, which primarily enters the body via mucosal surfaces and causes disease in the small intestines [17,18,19]. To enhance the mucosal immune response to PEDV, we selected a spike (S) of PEDV as a target to express [20,21]. The S protein contains major antigenic determinants and the core neutralising epitope (COE), which might be useful in preventing PEDV infection [22,23]. To effectively deliver the antigen into the small intestine, we engineered *B. subtilis* to express the recombinant PEDV COE region of the S protein and L-Lectin-β-GF to enhance the mucosal immunity of mice by increasing their ability to bind to M cells. This modified *B. subtilis* to express a PEDV antigen can also be used as a mucosal vaccine delivery system to effectively defend PEDV intestines infection.

## 2. Materials and Methods

### 2.1. B. subtilis, Plasmids and Animals

The *pHT43* plasmids and *B. subtilis* WB800N were given to Prof. Xuewen Gao, while PEDV SD-M was kindly presented by the Jiangsu Academy of Agricultural Sciences to our lab. The L-Lectin-β-GF sequence was synthesised according to the DNA sequences (GenBank, the accession number of YP_501439.1) and cloned into pET28a. Four to six-week-old SPF C57BL/6 and six to eight-week-old SPF Balb/C mice were bought from Comparative Medical Center of Yangzhou University. Vero cells were cultured in DMEM supplemented with 10% fetal bovine serum, 16 mM HEPES and 100 μg/mL penicillin/streptomycin (Life Technologies, Shanghai, China), which were then incubated in an atmosphere of 5% CO_2_ at 37 °C.

### 2.2. The Construction of Recombinant B.S.-RC and B.S.-RCL

In order to obtain recombinant *B. subtilis* WB800N, we firstly amplified three fragments. The RFP and COE fragments were cloned from *pDsRed-monomer-N1* and PEDV, while the L-Lectin-β-GF fragment was cloned from pET28a-L-Lectin-β-GF. Secondly, the above three fragments were overlapped to generate fusion fragments RFP-COE (RC) and RFP-COE-L-Lectin-β-GF (RCL). Thirdly, the fragments were insert into *pHT43* to generate *pHT43-RC* and *pHT43-RCL* by infusion clone, respectively. Finally, we transformed plasmids *pHT43-RC* and *pHT43-RCL* into WB800N, which were then named *B.S.-RC* and *B.S.-RCL*, respectively [24].

### 2.3. Analysis of Fusion Protein

LB medium with 5 μg/mL chloramphenicol were used to culture *B.S.-RC* and *B.S.-RCL*. We then added IPTG (0.1M) when the growth phase of recombinant *B. subtilis* reached an OD_600_ = 0.5 and continued the culture for another 3 h. After collecting, *B.S.-RC* and *B.S.-RCL* were washed three times with PBS and split by ultrasonication. Moreover, the rabbit anti-PEDV were selected to evaluate their expression by Western blot as previously described [25]. Finally, we used the Super ECL Plus system to visualise the protein bands.

### 2.4. Ligated Loop Experiments

**Figure 1:**
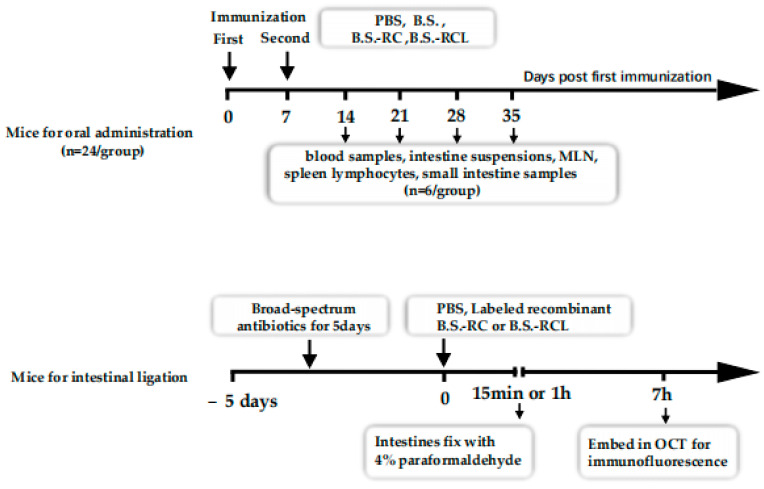
Immunisation and sample collection scheme.

SPF mice were treated with broad-spectrum antibiotics for 5 days before the experiments. Then, the mice were anesthetised with chloral hydrate according to their weight (350 mg per kg weight, intraperitoneal). After anesthetisation, terminal ileal or jejunal containing a Peyer’s patch ligated loop was injected into DyLight 488-labeled recombinant *B.S.-RC* and *B.S.-RCL* (200 mL) or the same volume of PBS (0.01 M) for 15 min or 1 h, as shown in Figure 1. The intestines were removed and fixed with 4% paraformaldehyde at 4 °C for 6 h and subsequently embedded in OCT (Sakura, Torrance, CA, USA) to cut into 8 μm for immunofluorescence [25]. The division can be seen in Table 1 for grouping.

Mice intestinal ligation and oral immunisation experiments were performed. The treatment and sample collections were conduct similar to the above scheme.

### 2.5. Immunisation Schedule and Samples Collection

C57BL/6 mice were immunised orally (*n* = 24 per group) at 0 and 7 days with *B.S.-RC* and *B.S.-RCL* at the concentration 10^10^ CFU/kg. Nonimmunised control mice were orally administrated 150 μL of 0.01 M PBS. The mice were sacrificed 14, 21, 28 and 35 days after first immunisation. The division can be seen in Table 1 for the grouping. Firstly, blood samples were taken and allowed to clot overnight at room temperature before collecting the serum for PEDV-specific IgG detection. The small intestine samples were washed with 0.5 mL of 0.01 M PBS, and the suspension was centrifuged and collected for PEDV-specific SIgA detection [2]. Secondly, small intestine containing Peyer’s patches tissue samples were fixed with 4% paraformaldehyde for immunofluorescence after 14 days of immunisation. In the meantime, intestine suspensions were collected for the total SIgA detection, while mesenteric lymph nodes (MLN) and spleen lymphocytes were isolated to assess the CD4^+^ and CD8^+^ T-cell populations.

### 2.6. Immunofluorescence Staining

For M cell staining, the slides were first washed with PBS three times to eliminate the residual OC. Then, the slides were blocked with PBS at room temperature for 30 min, which contained 5% FBS. Moreover, UEA-1-FITC was added and incubated at room temperature for 2 h to dye the M cells, while DAPI was used to stain the cell nuclei. Lastly, we used a Zeiss LSM710 confocal microscope to visualise the staining, and the images were analysed with the guidance of the regional Ethics Committee for Animal Testing (CREEA) (Permit Number No. 69387487) [5].

### 2.7. Enzyme-Linked Immunosorbent Assay

Nonspecific SIgA and PEDV-specific IgG and SIgA were measured by ELISA according to the instructions as previously described [26]. Briefly, the ELISA plates were first coated with PEDV COE protein at 4 °C overnight and saturated with PBS (1% BSA) at 37 °C for 2 h. Second, the mucosal suspension and serum were added and incubated for another 1 h at 37 °C. Subsequently, HRP-conjugated goat anti-mouse IgG and goat anti-mouse IgA were added and incubated at 37 °C for 1 h. Third, a substrate solution containing H_2_O_2_ and o-phenylenediamine was added at room temperature for 15 min before termination. Finally, we used an automated ELISA reader (Molecular Devices, Shanghai, China) to detect the absorbance at OD_450_ nm.

### 2.8. Statistical Analysis

All the results were analysed with GraphPad Prism 8.0 and expressed as the mean ± SD. Then, one-way analysis of variance was employed to evaluate the significant differences, followed by Dunnett’s *t*-test to evaluate the variations between the different groups. Differences were considered to be statistically significant when *p* < 0.05. Data were collected and combined from at least three independent experiments.

## 3. Results

### 3.1. Construction of Recombinant Plasmids and Fusion Protein Expression

**Figure 2:**
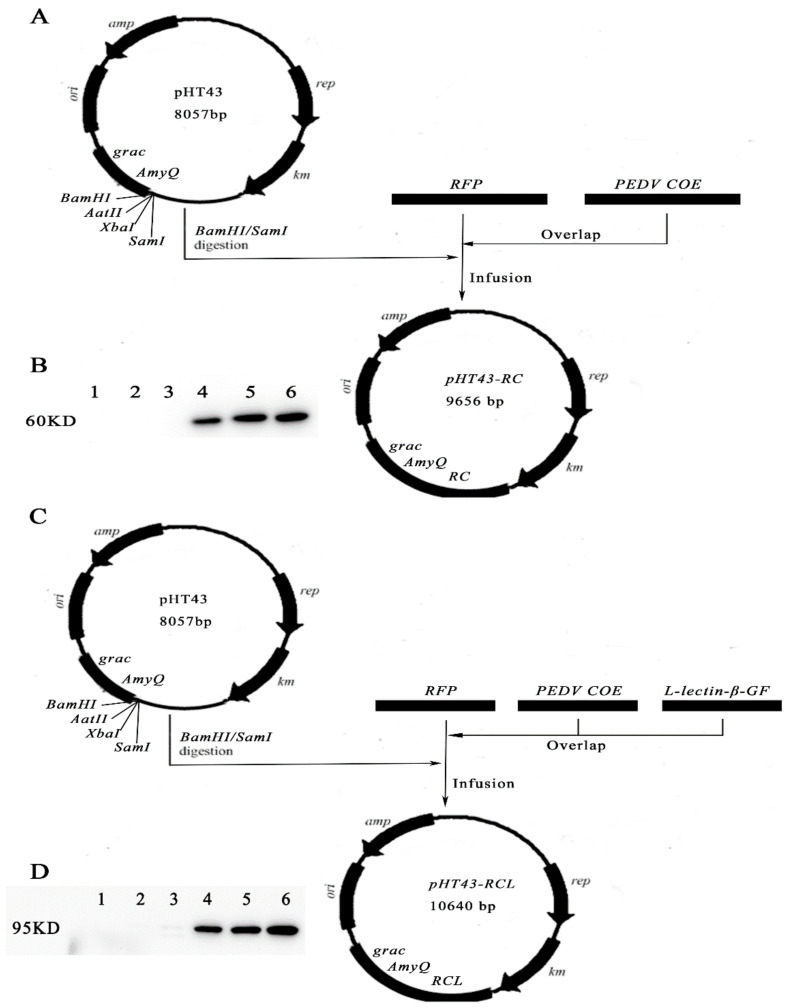
The construction of the recombinant plasmid and its fusion protein expression. (**A**) The RFP and PEDV S COE gene fragments were overlapped and inserted into *pHT43* to generate *pHT43*-RC. (**B**) Western blotting detected the recombinant RFP-COE protein (lanes 1, 2 and 3; *B. subtilis* WB800N; lanes 4, 5 and 6 and *B. subtilis* RC). The protein bands were approximately 60 kDa, expected at the size of fusion RFP-COE. (**C**) The RFP, PEDV S COE gene and L-lectin-β-GF fragments were overlapped and inserted into *pHT43* to generate *pHT43*-RCL. (**D**) Western blotting detected recombinant RFP-COE- L-lectin-β-GF (lanes 1, 2 and 3; *B. subtilis* WB800N; lanes 4, 5 and 6 and *B. subtilis* RCL). Protein bands were approximately 95 kDa, expected at the size of the fusion RFP-COE- L-lectin-β-GF, were detected.

The DNA fragments of the fusion proteins red fluorescent protein (RFP)-COE (RC) and RFP-COE-L-lectin-β-GF (RCL) were inserted into the *pHT43* plasmid to generate the *pHT43-RC* and *pHT43-RCL* shuttle vectors (Figure 2A,C). Then, the *pHT43-RC* and *pHT43-RCL* plasmids were transformed into *B. subtilis* WB800N by electroporation to generate strains *B. subtilis*-RC (*B.s-RC*) and *B. subtilis*-RCL (*B.s-RCL*). The protein expression was evaluated in *B.s-RC* and *B.s-RCL* via Western blotting using anti-PEDV antibodies (Figure 2B,D). Clear, positive bands were present at 60 and 95 kDa in recombinant strains *B.s-RC* and *B.s-RCL*, respectively (Supplementary Materials Figures S1 and S2). Our results showed that the RC and RCL fusion proteins were successfully expressed in *B. subtilis* WB800N.

### 3.2. Animal Experiments Design and sIgA Levels and M Cell Numbers in Mouse Intestines

**Figure 3:**
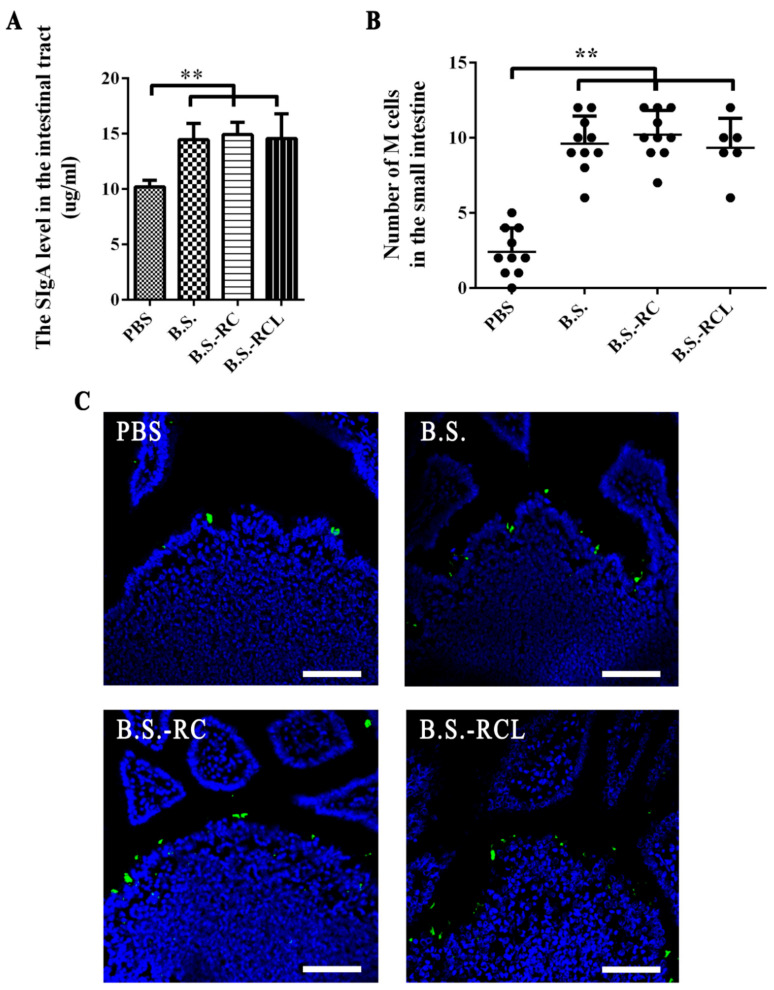
Analysation of the SIgA level and M cell number in the mice intestine. (**A**) The SIgA level in the mice intestine were displayed by histogram (Signifcance diference was expressed as, ** *p* < 0.01). Orally administrated with PBS, *B. subtilis* WB800N, *B. subtilis* RC and *B. subtilis* RCL at 0 and 7 days and the sample were collected at 14, 21 and 28 days. M cells were stained by UEA-1. (**B**) The number of M cells in the small intestine of each mouse was displayed. (**C**) Immunofluorescence staining results of M cells in mice small intestines (oral PBS group, oral *B. subtilis* WB800N group, oral *B. subtilis* RC group and oral *B. subtilis* RCL group. Scale bars = 50 μm).

Our animal experiment was divided into two parts, which were the ligated loop experiments and animal immune experiment. The immunisation and sample collection scheme are clearly shown in Figure 2. Since sIgA can interact specifically with mucosal M cells in gut-associated lymphoid tissues [16], we therefore examined the sIgA levels and M cell numbers in mouse intestines following the oral treatment with the recombinant *B. subtilis*. sIgA levels increased in the intestinal tracts of the mice at 14 days after feeding with *B. subtilis* (Figure 3A). Moreover, we stained the mouse intestinal M cells 14 days after the first immunisation; the number of M cells in the mouse intestines increased dramatically after feeding with recombinant *B. subtilis* (Figure 3B,C).

### 3.3. Co-Localisation of M Cells and Recombinant B. subtilis

**Figure 4:**
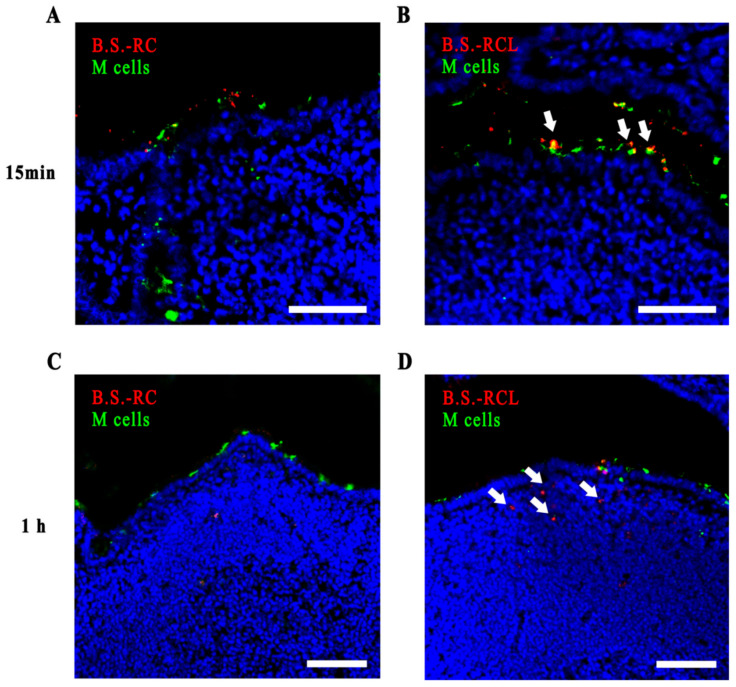
The observation of co-located M cells and recombinant *B. subtilis*. The ligated loops of mice were injected with Dylight 488-labelled *B. subtilis* RC and Dylight 488-labelled *B. subtilis* RCL for 15 min and 1 h, respectively. Frozen sections of the ileum were stained with UEA-1 (green) and DAPI (blue). (**A**,**B**) The co-located M cells and Dylight 549-labelled *B. subtilis* RC or *B. subtilis* RCL (white arrows) 15 min after recombinant *B. subtilis* injection. (**C**,**D**) The co-located M cells and Dylight 549-labelled *B. subtilis* RC or *B. subtilis* RCL (white arrows) 1 h after recombinant *B. subtilis* injection. Scale bars = 10 μm.

M cells are derived from stem cells located within intestinal crypts [27], which can be stimulated by the activator of nuclear factor kappa-Β ligand (RANKL) secreted by subepithelial stromal cells [28]. The L-lectin-β-GF protein binds to sIgA and M cells [15]. To investigate the binding of recombinant *B. subtilis* and M cells in vivo, we performed ligated loop experiments in mice. We found that *B.s*-*RCL* localised to M cells 15 min after the oral administration. Moreover, the number of *B. subtilis* cells in the follicular-associated epithelium (FAE) was higher in mice fed with recombinant *B.s-RCL* than in those fed with control *B. subtilis* at 1 h after administration. Our results suggested that recombinant *B.s*-*RCL* effectively adhered to the M cells (Figure 4A,B) and increased the amount of recombinant *B. subtilis* in the FAE (Figure 4C,D).

### 3.4. Systemic and Local Immune Responses after Oral Immunisation

**Figure 5:**
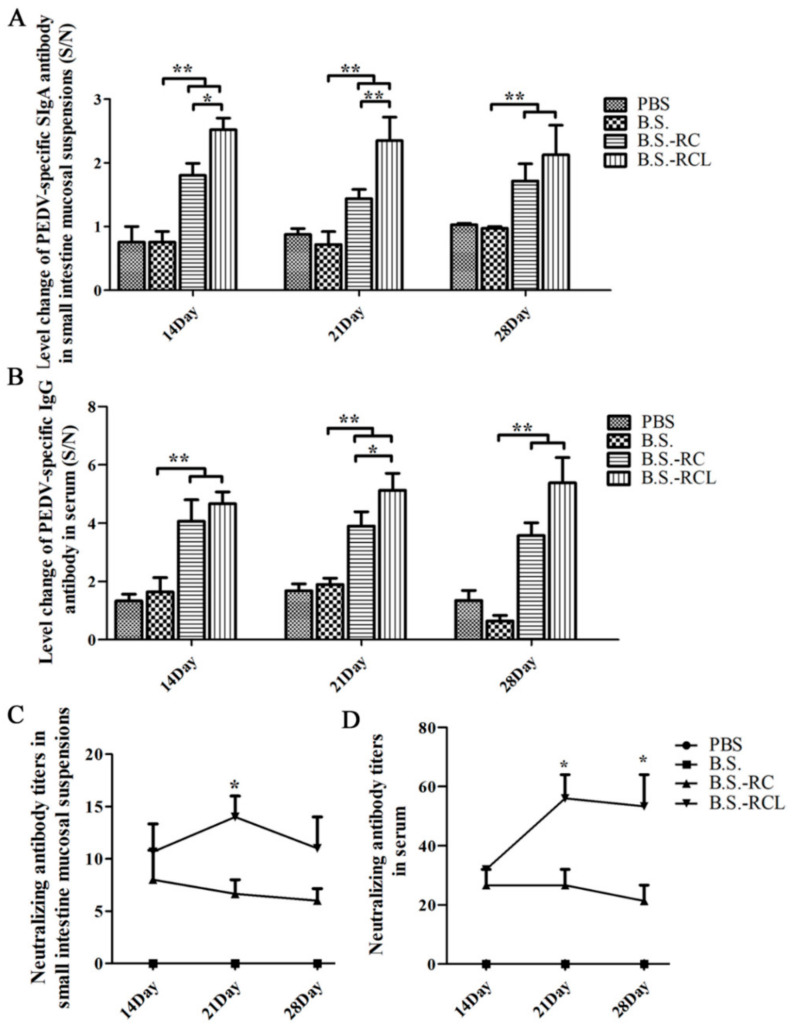
The detection of specific antibody levels after oral immunisation. After 14, 21 and 28 days of immunisation, the samples were collected to analyse PEDV-specific SIgA (**A**), IgG (**B**) and neutralising antibodies in small intestine mucosal and serum (**C**,**D**) (the value of S/N was calculated) by indirect ELISA (*n* = 6). Data are shown as the mean ± SD of three samples. *, *p* < 0.05; **, *p* < 0.01.

**Figure 6:**
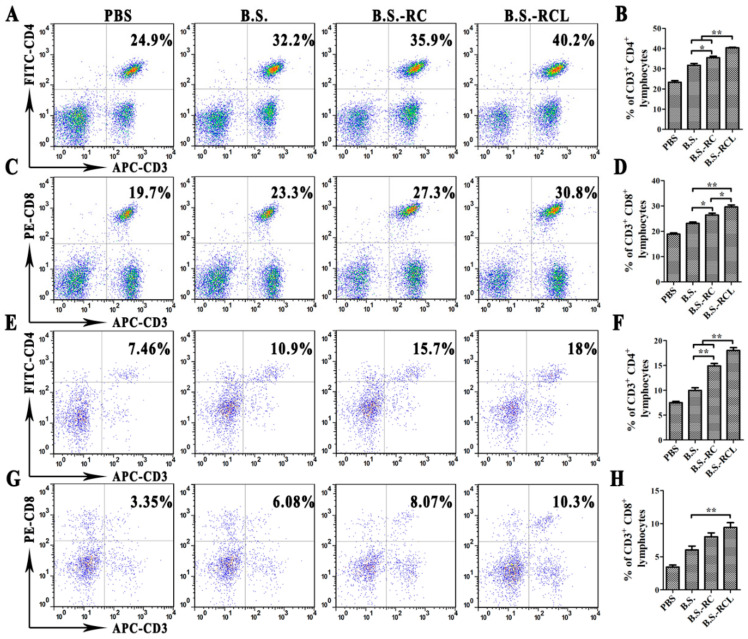
The changes of the local and systemic cellular immunity after oral immunisation. The MLN and spleen collected from mice 14 days after immunisation, and then, the percentages of CD3^+^ and/or CD4^+^ in MLN (**A**,**B**), CD3^+^ and/or CD8^+^ in MLN (**C**,**D**), CD3^+^ and/or CD4^+^ in the spleen (**E**,**F**) and CD3^+^ and/or CD8^+^ in spiralising (**G**,**H**) T cells were analysed by FACS (Signifcance diference was expressed as, * *p* < 0.05, ** *p* < 0.01).

Antigen-specific sIgA is a key player in mucosal immunity. Therefore, we hypothesised that the sIgA-binding L-lectin-β-GF protein could enhance the carrier function and adjuvant properties of *B. subtilis*. We detected PEDV-specific sIgA (Figure 5A) at 14–28 days after recombinant *B. subtilis* oral administration. Furthermore, we found that the levels of PEDV-specific sIgA were higher in mice orally administered *B.s*-*RCL* compared to those administered *B.s*-*RC* at 14–21 days after administration. Mice administered recombinant *B. subtilis* exhibited higher PEDV-specific immunoglobulin G (IgG) antibody titres compared to those administered controls of *B. subtilis* at 14–28 days after administration (Figure 5B,C). Similarly result of serum antibody titres were also found enhanced by recombinant *B. subtilis* stimulation (Figure 5D). In addition, we found that the percentages of CD3^+^/CD4^+^ and CD3^+^/CD8^+^ T cells in mesenteric lymph nodes (MLNs) and spleen lymphocytes were markedly higher after *B.s*-*RCL* stimulation (Figure 6). These results indicate that oral immunisation with recombinant *B.s*-*RCL* effectively induced systemic and local immune responses in mice.

## 4. Discussion

*B. subtilis* is commonly used to express heterologous proteins and as an antigen delivery vector [5,29,30,31]. The spores formed by *B. subtilis* have unique resistance properties, enabling their survival under extreme conditions. *B. subtilis* is also used as a feed additive to improve growth and stimulate the immune responses of livestock [32]. Orally administrated *B. subtilis* exerts beneficial effects on intestinal lymphoid tissue in chickens [1]. In this study, orally administrated *B. subtilis* increased intestinal M cells in Peyer’s patches in mice. Although the underlying mechanisms are still unclear, *B. subtilis* have probably adjuvant effects due to its ability to produce amylase, protease, lipase and amino acids [33]. M cells are primarily derived from stem cells located within dome-associated crypts [27] but also originate from stimulated enterocytes. The increase in M cells likely increased the number of recombinant *B. subtilis*-binding events while also enhancing the uptake and delivery of antigens.

Recently, several oral vaccines have been developed. Oral vaccines are particularly effective for building defences against mucosal pathogens. Various studies have focused on enhancing antigen deliveries to M cells due to their role in antigen capturing. Some specialised proteins, such as invasion, bind to the surface receptors of M cells to enter the intestine [34,35]. Besides the uptake and transfer of antigens, M cells are also involved in sIgA responses [36]. Hence, we expressed the M cell-targeting protein L-lectin-β-GF to mediate interactions with M cells and trigger sIgA secretion. Our intestinal ligated loop mouse experiments demonstrated that *B.s*-*RCL* associated with M cells after 15 min. Furthermore, they revealed that L-lectin-β-GF can enhance the delivery of *B. subtilis*, demonstrating a novel mucosal immune delivery system. Our findings were similar to those of Rochereau et al., who found that the p24 protein could be employed as a vehicle to induce humoral and cellular immune responses against human immune deficiency virus [37].

PEDV is a serious swine intestinal disease; infections occur at the mucosal surfaces, especially those of the intestinal mucosal epithelium. PEDV entry could be prevented by a well-established mucosal sIgA immune response. Here, we found that *B. subtilis* treatment increased the sIgA levels in the intestinal tracts of mice, which enhanced the binding of L-lectin-β-GF to M cells, thus further improving the mucosal immunity. Specific sIgAs effectively neutralise viruses. Following the oral administration of *B.s-RC*, the PEDV-specific sIgA levels increased dramatically. Mice administered *B.s-RCL* exhibited higher levels of PEDV-specific sIgA at 14 days after the first immunisation.

In this study, we employed *B. subtilis* as a vehicle to express the PEDV S protein COE and L-lectin-β-GF protein. Our work suggests that recombinant *B. subtilis* may promote growth and regulate immune responses in piglets while also targeting and binding to M cells to enhance antigen presentation and augment defences against PEDV invasion. However, we did not perform the in vitro M cell experiment to demonstrate that L-lectin-β-GF could enhance the M cell-mediated phagocytic for the lack of a M cell model. This study provided important insights that could facilitate the development of a PEDV vaccine. We demonstrated that immunising mice with *B.s*-*RCL* significantly increased faecal PEDV-specific sIgA titres and serum IgG levels.

## 5. Conclusions

Bacillus subtilis, as a feeding additive, has been widely used for heterologous antigen expression in immune mucosal. Hence, our study tried to defend PEDV by expressing a PEDV antigen with B. subtilis. Initially, we constructed the recombinant B. subtilis strain (B.s-RCL) to induce the mucosal secretory immunoglobulin A (sIgA) and anti-PEDV serum immunoglobulin G (IgG) production, as well as to increase the number of microfold cells (M cells). Then, oral immunisation mice with B.s-RCL not only increased PEDV-specific sIgA in the small intestinal but also upregulated the percentages of CD4^+^ and CD8^+^ T cells in mesenteric lymph nodes and the spleen. All in all, our study developed a novel oral vaccine to prevent porcine diarrhoea epidemic.

## Figures and Tables

**Table 1:** Experimental groups and administration strategies.

	Groups	Treatments
Intestinal ligation	PBS-15 min	PBS (200 mL for 15 min or 1 h)
	PBS-1 h	
	*B.S.-RC*-15 min	DyLight 488-labeled recombinant *B.S.-RC* (200 mL for 15 min or 1 h)
	*B.S.-RC*-1 h	
	*B.S.-RCL*-15 min	DyLight 488-labeled recombinant *B.S.-RCL* (200 mL for 15 min or 1 h)
	*B.S.-RCL*-1 h	
Oral administration	PBS	PBS
	PBS (0.01M PBS 150 μL, *n* = 24)	PBS (0.01 M PBS 150 μL, *n* = 24)
	B.S.	B.S.
	B.S. (1010 cfu/kg)	B.S. (1010 cfu/kg)

## Data Availability

All data generated or analysed during this study are included in this published.

## Supplementary Materials

The following supporting information can be downloaded at: https://www.mdpi.com/article/10.3390/vetsci9050211/s1, Figure S1: Western blot result of the fusion protein *B. subtilis* RC.; Figure S2: Western blot result of the fusion protein *B. subtilis* RCL.
