# Supplementary material for: Immune Responses Induced by Recombinant Bacillus subtilis Expressing the PEDV Spike Protein Targeted at Microfold Cells
Source: Vet Sci. 2022 Apr 25;9(5):211. doi: 10.3390/vetsci9050211 (PMC9143571; doi:10.3390/vetsci9050211)
Supplement: Supplementary file 1 [file vetsci-09-00211-s001.zip › vetsci-1675854-supplementary.pdf]

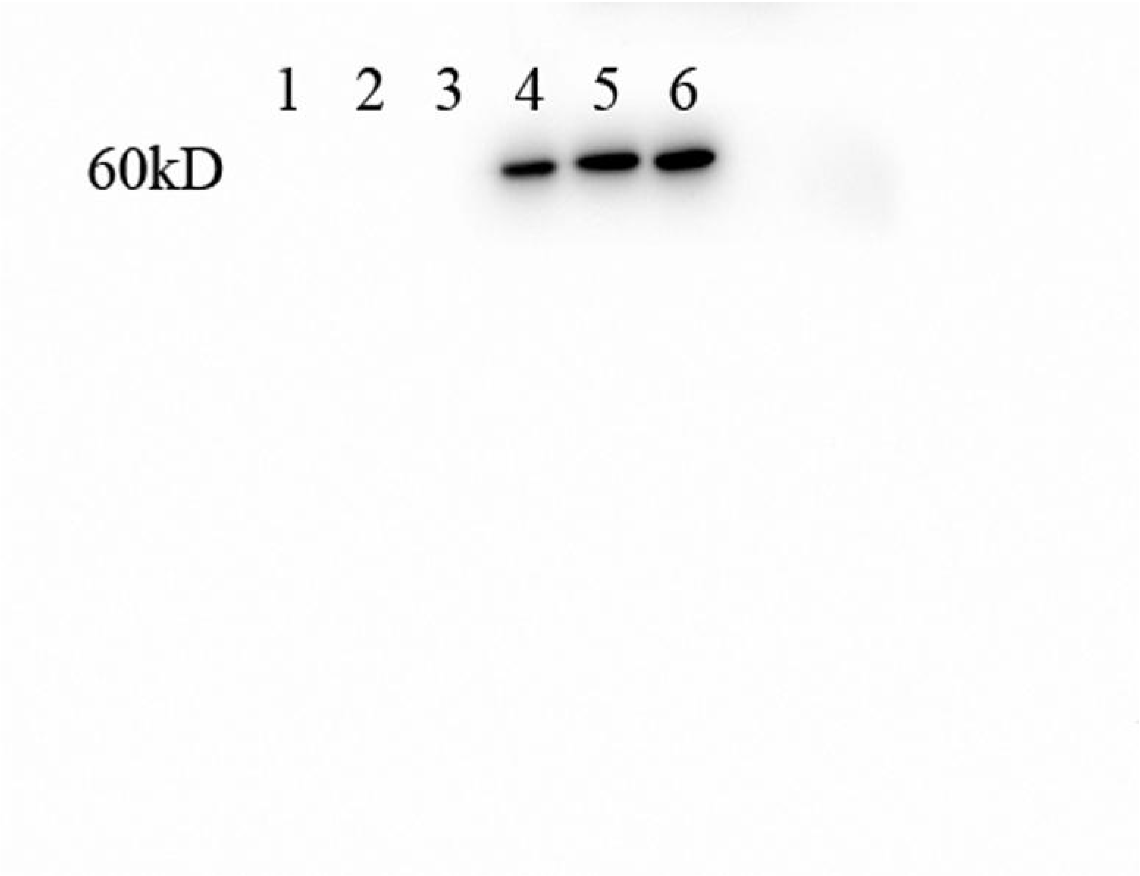

Figure S1. Western blot result of the fusion protein B. subtilis RC.

Western blotting detect recombinant RFP-COE protein (lane 1, 2, and 3, B. subtilis WB800N; lane 4, 5, and 6, B. subtilis RC). Protein bands were approximately 60 kDa, expected to the size of fusion RFP-COE.

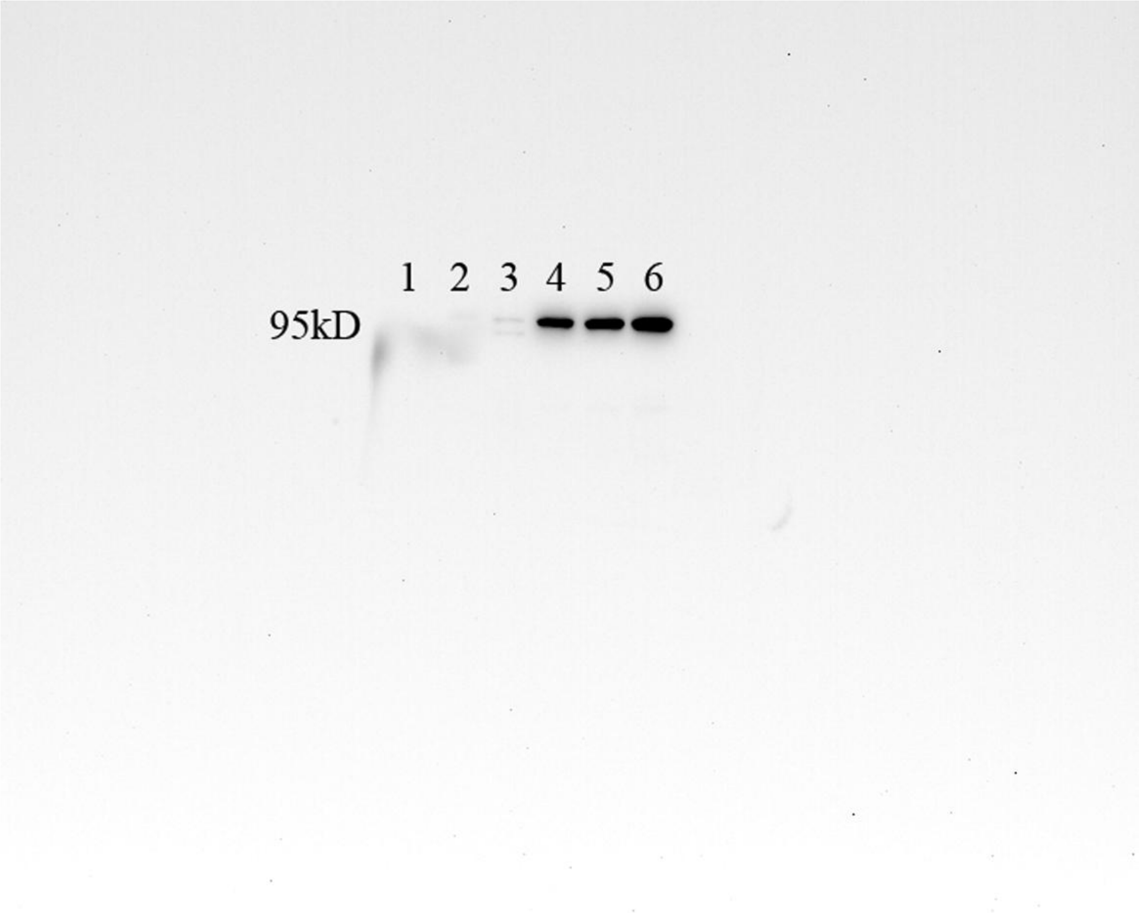

Figure S2. Western blot result of the fusion protein B. subtilis RCL.

Western blotting detect recombinant RFP-COE- L-lectin-β-GF (lane 1, 2, and 3, B. subtilis WB800N; lane 4, 5, and 6, B. subtilis RCL). Protein bands were approximately 95 kDa, expected to the size of fusion RFP-COE- L-lectin-β-GF, were detected.
